# *Pseudosulfitobacter pseudonitzschiae* hitchhikes on gliding colonies of *Cellulophaga lytica*

**DOI:** 10.1093/ismeco/ycaf118

**Published:** 2025-07-16

**Authors:** Asimenia Gavriilidou, Maria Murace, Marina Portoghese, Sanne Schouten, Raditijo Hamidjaja, Álvaro Escobar Doncel, Sjef Boeren, Marcel Giesbers, Jérémie Capoulade, Silvia Vignolini, Hauke Smidt, Colin J Ingham

**Affiliations:** Laboratory of Microbiology, Wageningen University & Research, Stippeneng 4, 6708WE, Wageningen, The Netherlands; Yusuf Hamied Department of Chemistry, University of Cambridge, CB2 1EW, Cambridge, United Kingdom; Yusuf Hamied Department of Chemistry, University of Cambridge, CB2 1EW, Cambridge, United Kingdom; Hoekmine BV, Verenigingstraat 36, 3515GJ, Utrecht, The Netherlands; Hoekmine BV, Verenigingstraat 36, 3515GJ, Utrecht, The Netherlands; Hoekmine BV, Verenigingstraat 36, 3515GJ, Utrecht, The Netherlands; Laboratory of Biochemistry, Wageningen University & Research, Stippeneng 4, 6708WE, Wageningen, The Netherlands; Wageningen Electron Microscopy Centre, Wageningen University & Research, Droevendaalsesteeg 1, 6708PB, Wageningen, The Netherlands; Department of Bionanoscience, Kavli Institute of Nanoscience, Delft University of Technology, van der Maasweg 9, 2629 HZ, Delft, The Netherlands; Yusuf Hamied Department of Chemistry, University of Cambridge, CB2 1EW, Cambridge, United Kingdom; Sustainable and Bio-inspired Materials, Max Planck Institute of Colloids and Interfaces, Potsdam 14476, Germany; Laboratory of Microbiology, Wageningen University & Research, Stippeneng 4, 6708WE, Wageningen, The Netherlands; Sustainable and Bio-inspired Materials, Max Planck Institute of Colloids and Interfaces, Potsdam 14476, Germany

**Keywords:** microbial interactions, structural colour, motility, quorum sensing, coculture, proteogenomics, optics

## Abstract

Interspecies interactions shape microbial communities; this is central for microbial ecology. *Cellulophaga lytica* PlyA2 is a marine flavobacterium, which glides over surfaces and forms ordered, structurally coloured colonies, which display angle-dependent reflection of light. *Pseudosulfitobacter pseudonitzschiae* SW is an apparently nonmotile, nonstructurally coloured marine bacterium. Here, we aim to understand the interaction of both strains at cellular, genomic, optical, and proteomic levels. Cocultivation on agar showed that *P. pseudonitzschiae* uses gliding *C. lytica* to spread by microbial hitchhiking in which *Pseudosulfitobacter* appears to “surf” on basal layers of motile *Cellulophaga*. This dispersal mechanism was found to be often beneficial for *P. pseudonitzschiae,* which could maximally expand its population up to 350-fold relative to monoculture. Coculture was often of limited benefit for *C. lytica*, only in extended cultivation on rich medium was the presence of *P. pseudonitzschiae* detrimental to its viability. The proteome of *P. pseudonitzschiae* was strongly impacted by the association with *C. lytica*. Quorum-sensing signalling, potential exchange of amino acids, vitamins, and other metabolites are likely mediating this hitchhiking interaction. In contrast, *C. lytica* made minimal adjustments to its proteome composition in coculture. Supported by optical analysis, *P. pseudonitzschiae* patterned *C. lytica* by changing how groups of the latter organised to reflect light. Our results underscore the unusual, dynamic interplay between two bacterial species and provide insights on the mechanisms underlying this relationship.

## Introduction

A myriad of interactions between environmental bacteria, from cooperative to competitive, contribute to microbial distribution [1]. Bacterial motility mediates many of these interactions [2] and groups of motile bacteria can move sessile microorganisms. This process has been described as transport [3, 4] or hitchhiking [5, 6]. The sessile partner gains by dispersal and often gives benefits to the transporting bacteria, such as nutrition or antibiotic resistance [3]. Hitchhiking has been shown to be associated with swarming [4], swimming, [5] and gliding bacteria [7, 8]. For example, *Capnocytophaga gingivalis* (class *Flavobacteriia*) is found in the oral cavity and can glide and redistribute, and therefore pattern, nonmotile bacterial species from the same ecosystem [8]. Also, motile, spherical aggregates of *Flavobacterium johnsoniae*, termed zorbs, collect and transport bacteria from other species [9].

*Cellulophaga lytica* PlyA2 is a marine, gliding bacterium, member of the *Flavobacteriia* [10]. As with *C. lytica* CECT 8139 [11, 12] and DSM7489 [13], cells align in colonies to form a polydomain 2D crystalline arrangement [10] leading to vivid, angle-dependent structural colour (SC) when illuminated. SC is an optical phenotype widespread in the tree of life, including many *Flavobacteriia* [10, 14–18]. Formation of SC is facilitated by gliding motility; nonmotile mutants of *C. lytica* CECT 8139 appear dull, indicating a reduced capacity for SC [12, 14, 19]. Colonies of another SC-forming *Flavobacterium*, strain IR1, with transposon insertions in genes for gliding motility also appear dull [12, 17]. Although the biological significance of bacterial SC remains unknown, it has been suggested that in strain IR1 the cell organization underlying the photonic crystalline arrangement is advantageous in competition with other bacteria [20].

Cocultures play fundamental roles in studying microbial interactions. Here, we set up two-species cocultures of the gliding, structurally coloured *C. lytica* and the nonmotile, non-SC bacterium *Pseudosulfitobacter pseudonitzschiae*. The aim of this study was to determine how the presence of one species affects the other and the role of each member in a bacterial consortium that might equally appear in nature. We describe a hitchhiking interaction on agar surfaces where *C. lytica* PlyA2 transports the nonstructurally coloured bacterium *P. pseudonitzschiae* SW. Our initial hypothesis was that their relationship is cooperative/mutualistic. Therefore, we determine which strain gains from this interaction under a variety of growth conditions. The complex relationship was further analysed both in terms of the resulting anatomy of the expanding colony and of its peculiar optical response. A proteogenomic approach was employed to investigate the effect of coculture on the proteomes of both partners. This work reveals an unusual asymmetric interaction that changes under different growth conditions and suggests that ecological interactions can be subtle and shifting.

## Materials and methods

### Culture conditions

Strains are as indicated in Table S1. Growth of *C. lytica* strains and *P. pseudonitzschiae* SW was at 28°C under aerobic conditions in 10 × 10 cm and Rich Marine (RMAR) agar (0.8% w/v) plates [10]. RMARLow was RMAR agar without peptone. Agar plates included black dye, nigrosine (0.05% w/v) for optical contrast. Selective viable counts were used to quantify the strains in coculture (Supplementary Methods).

### DNA sequencing and genome analysis

DNA extraction [21], genome sequencing [10], strain identification from 16S rRNA [20] were as described. Data have been deposited under accession PRJEB56913 at the European Nucleotide Archive. Workflows for genome assembly [22–26], metabolic pathway reconstruction [27–29] and functional annotation [30, 31] were as cited in the Supplementary Methods.

### Microscopy and image processing

Images were captured with Olympus BX-41 and Keyence VHX-7000 microscopes and quantified by ImageJ (v1.52) [32]. Confocal microscopy was performed on Syto9 stained agar blocks [20]. Scanning electron microscopy followed standard procedures [33]. Photography of colonies was as previously described [34].

### Proteomics

Strains were grown on RMAR agar, but using 1.2% (w/v) agar, omitting nigrosine, using four biological replicates for each condition (coculture and monocultures). After harvesting and cell lysis, sample preparation was done by protein aggregation capture [35, 36] (Supplementary Methods). Protein identification/quantification was performed by nano-liquid chromatography mass spectrometry (nLC100, Thermo Scientific, MA, USA) and MS/MSMS spectra were measured with an Orbitrap Exploris (Thermo Scientific, MA, USA) [37]. Raw LCMS-MS data were analysed with MaxQuant 2.0.3.0 [38] and PTXQC was used to check data quality [39]. Analysis of the processed proteomics data was performed in Rstudio [40] using R 4.3.2 [41]. Differences in protein abundance (coculture vs monoculture) were deemed significant if |log_2_FC| ≥1.5 and adjusted *P*-value ≤.05. Details on proteomics data analysis and visualization are in the Supplementary Methods.

### Angle-resolved optical spectroscopy

Reflectance spectra for the analysis of SC were acquired at different angles of incidence and detection using an in-house built angle-resolved spectrometer as described [17, 33].

## Results

### *Pseudosulfitobacter pseudonitzschiae* modulates the abundance and structural colour of *Cellulophaga lytica* on agar plates

**Figure 1:**
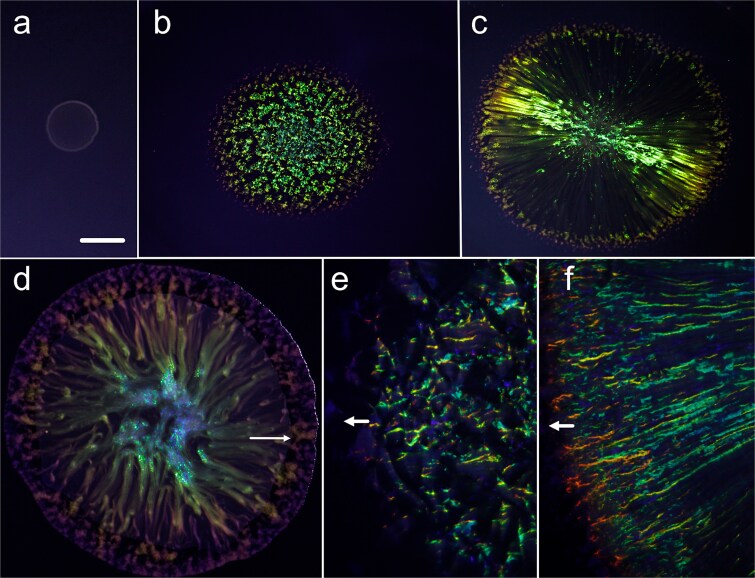
Expanding colonies of C. *l*ytica PlyA3 and *P. pseudonitzschiae* SW inoculated into the centre of agar plates and cultivated for 3 days at 28°C. (a) *Pseudosulfitobacter pseudonitzschiae* SW cultivated on RMAR agar showing a nonspreading, nonstructurally coloured colony. (b) An expanding colony of *C. lytica* PlyA3 on RMAR agar. The angle of photography and illumination were optimal for viewing SC. (c) As panels a and b, but with *P. pseudonitzschiae* and *C. lytica* PlyA3 coinoculated in the centre of the colony. The SC is from *C. lytica*. (d) Both strains coinoculated on RMARL agar plates, viewed directly from above. Arrow shows the outer limit of *P. pseudonitzschiae.* (e) Detail of the edge of a colony of *C. lytica* PlyA3 cultured as panel b, showing pointillistic SC. Arrow shows the direction of colony expansion. (f) Detail of the edge of a colony of *C. lytica* PlyA3 and *P. pseudonitzschiae*, cocultured as panel c, showing radially patterned SC. Arrow shows the direction of colony expansion. The scale bar (panel a) indicates 1 cm when applied to panels a–d and 0.4 mm when applied to panels e and f.

*Pseudosulfitobacter pseudonitzschiae* SW formed nonspreading colonies when inoculated into the centre of rich (RMAR) or limited (RMARLow) nutrient agar plates and incubated at 28**°**C for 3 or 10 days. SC was not observed for *Pseudosulfitobacter*, as judged by viewing colonies on plates containing nigrosine, with illumination with a white LED (Fig. 1a). However, gliding *C. lytica* PlyA2 colonies inoculated onto either culture media expanded at up to 12 mm/day and displayed intense, pointillistic SC (Fig. 1b and 1e). To observe interspecies interactions, mixed cultures were co-inoculated on RMAR and RMARLow agar plates, then incubated using the same conditions as in the monocultures. Cocultivation of PlyA2 and SW resulted in colonies expanding at 12–13 mm/day. After 3 days on RMAR agar, cocultured colonies had a slightly greater diameter than *C. lytica* PlyA2 in monoculture. Cocultivation (3 days) resulted in colonies with a radial patterning of SC that was different to the patterning of *C. lytica* PlyA2 when grown alone (Fig. 1c, d, and  f). This radial patterning was lost at the colony periphery (Fig. 1c). Viewing cocultured colonies, from directly above (Fig. 1d) revealed a matte effect across most of the colony, except at the periphery, coinciding with the radial patterning. As will be shown in later sections, radial patterning was the result of the interaction of *P. pseudonitzschiae* and *C. lytica*.

### Determining the distribution of *Pseudosulfitobacter pseudonitzschiae* and *Cellulophaga lytica* in cocultured colonies

**Figure 2:**
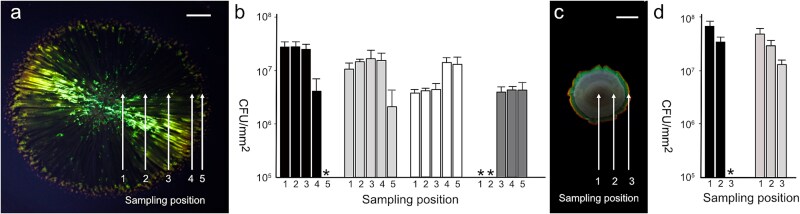
Mapping the distribution of *C. lytica* and *P. pseudonitzschiae* SW within mixed colonies by sampling and selective viable counts. (a) Expanding colony of *C. lytica* PlyA3 and *P. pseudonitzschiae* SW on RMAR (0.8% w/v) agar, after 3 days incubation at 28°C. Sampling points are marked from 1 (centre) to 5 (edge) of the colony. Scale bar (upper right) indicates 1 cm. (b) Selective viable counts from a sampled RMAR plate as panel a, showing average cfu (calculated as cfu/mm^2^), with error bars indicating the upper limit of S.D. above the mean. Black bars: *P. pseudonitzschiae* SW viable counts as sampled from the plate shown in panel a. **+** indicates no cfu were detected for these samples, indicating a viable count of <100 cfu/mm^2^. Light grey bars: cfu of *C. lytica* PlyA3. White bars: *P. pseudonitzschiae* SW sampled an RMAR plate similar to that shown in panel a but after 10 days. Dark grey bars: cfu of *C. lytica* PlyA3. * no cfu were detected for these samples, indicating a viable count of <100 cfu/mm^2^. (c) Colony after coculture of *C. lytica* PlyA4 (nongliding) and *P. pseudonitzschiae* SW for 3 days on RMAR. Scale bar indicates 0.8 mm. Three sampling points are indicated. (d) Selective viable counts from the colony shown in panel c. Black bars: *P. pseudonitzschiae* SW. **+** indicates no *P. pseudonitzschiae* cfu were detected, indicating a viable count of <100 cfu/mm^2^. Light grey bars: *C. lytica* PlyA4.

To map the distribution of viable bacteria in cocultured colonies, samples were taken at different locations followed by selective viable counts (Fig. 2a and b). To facilitate this, a spontaneous rifampicin resistant mutant of *C. lytica* (PlyA3) was isolated with SC, gliding motility and interactions in coculture identical to the parental strain PlyA2 (Table S1). After 3 days of coculture on RMAR agar, *P. pseudonitzschiae* SW was isolated from all sections of the colony (positions 1–4) except the edge (position 5). *Cellulophaga lytica* PlyA3 was found throughout the colony. After 10 days of coculture on RMAR agar, *P. pseudonitzschiae* was present throughout the colony with the viability declining by an order of magnitude in most of the colony (positions 1–3), but abundant towards the periphery of the plates (positions 4 and 5). After 10 days, viable *C. lytica* PlyA3 was not isolated from the same colony interior, (positions 1–2) but could be retrieved from positions 3 to 5. This distribution correlated with SC which, after 10 days, had been lost from locations 1 to 2 but was visible at positions 3 to 5. This contrasts with monoculture, under the same conditions, from which viable *C. lytica* could be recovered from position 1 (Table S2).

### Gliding motility by *Cellulophaga l*ytica is important in expansion and patterning of colonies when grown with *Pseudosulfitobacter pseudonitzschiae*

To determine if the observed phenotypes were related to the gliding of *C. lytica*, we repeated cocultivation experiments using a spontaneous mutant of strain PlyA3, *C. lytica* PlyA4, which was nonspreading or gliding on RMAR agar and showed reduced SC. Coculture of *C. lytica* PlyA4 and *P. pseudonitzschiae* SW (Fig. 2c) on RMAR agar gave smaller colonies than coculture with the wild type (WT) (Fig. 2a) with reduced SC and no radial patterning after 3 days. Mapping the distribution of the two strains in the colony indicated that after 3 days *C. lytica* PlyA4 colonies had a 3 mm ring of dull SC at the edge. Viable cells of *P. pseudonitzschiae* were not recovered from the colony edge (Fig. 2d), whilst at positions 1 and 2, in the colony interior, both species were recovered in similar numbers. It is concluded that gliding by *C. lytica* is important in the phenotypes of colony expansion and patterning of SC (Fig. 1). However, when cultivated alone, on RMAR agar, for 3 days, neither *C. lytica* PlyA4 nor *P. pseudonitzschiae* SW created colonies >8 mm diameter, yet the cocultured colonies were >31 mm diameter, suggesting interactions beyond the dispersal effect of gliding motility. As described in the next section, there was also an increase in the abundance of both species due to coculture, despite the loss of motility by the *Cellulophaga* strain.

### The *Pseudosulfitobacter pseudonitzschiae* population gains more than *Cellulophaga lytica* from cocultivation as determined by whole colony, selective, viable counts

**Figure 3:**
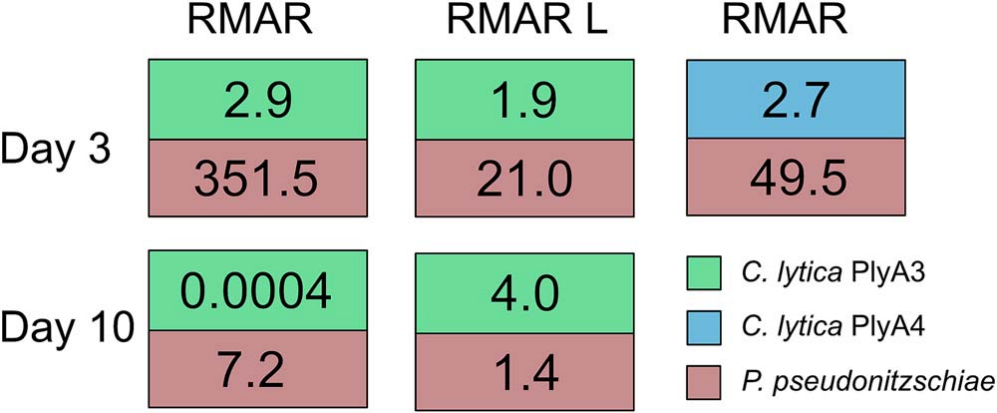
The effect of coculture on the total viable populations of both species. Viable counts for whole colonies of *C. lytica* PlyA3 or PlyA4 and *P. pseudonitzschiae* SW, cultured both individually and together under different conditions. The numbers represent the ratio of total viable counts in coculture and monoculture for each strain (Table S2). Values above 1 indicate a beneficial relationship, i.e. a particular strain increased its population due to associating with the other strain. Values below 1 indicate antagonism. For example, Day 10 for *C. lytica* on RMAR, a value of 0.0004 indicates the viability of this strain was decreased by the presence of *P. pseudonitzschiae* SW, whilst the latter gained over 7-fold.

We estimated the effect of coculture on the total viable population of both species in entire colonies (Table S2). The ratio of viable cell counts (coculture:monoculture) was determined for each strain under different growth conditions, rich and low in nutrients agar (Fig. 3). In most situations the two strains had a beneficial association (a ratio of viable cells, coculture:monoculture >1). *Pseudosulfitobacter pseudonitzschiae* SW showed a >350-fold increase of its population in the presence of the gliding *C. lytica* PlyA3 on RMAR agar medium, compared to the *P. pseudonitzschiae* SW monoculture, after 3 days. Population gains for *P. pseudonitzschiae* SW, cultured with *C. lytica* PlyA3, also occurred after 10 days on the high nutrient RMAR agar but was reduced in magnitude (ratio of 21, Fig. 3). In addition, the nonmotile *C. lytica* PlyA4 stimulated a population increase in *P. pseudonitzschiae* SW (ratio 49.5) (RMAR, 3 days), but less than for the parental gliding strain. Stimulation of *P. pseudonitzschiae* SW growth by *C. lytica* PlyA3 also occurred on the RMARLow agar but this was reduced (7.2-fold after 3 days, 1.4 at 10 days). *Cellulophaga lytica* PlyA3 and PlyA4 growth was stimulated only moderately by the presence of *P. pseudonitzschiae* SW (ratios 1.9 to 4). The exception was after 10 days on RMAR agar when *P. pseudonitzschiae* SW proved inhibitory to *C. lytica* PlyA3, decreasing the viable cells of *C. lytica* PlyA3 by over three orders of magnitude (Fig. 3) compared to a 4-fold decrease in the viability of PlyA3 alone in the same period. In summary, *P. pseudonitzschiae* SW gained from the association with *C. lytica* under all conditions tested. *C. lytica* gained considerably less and was notably disadvantaged by the association after 10 days on rich media.

### Nonmotile *Pseudosulfitobacter pseudonitzschiae* hitchhikes on *Cellulophaga lytica*

**Figure 4:**
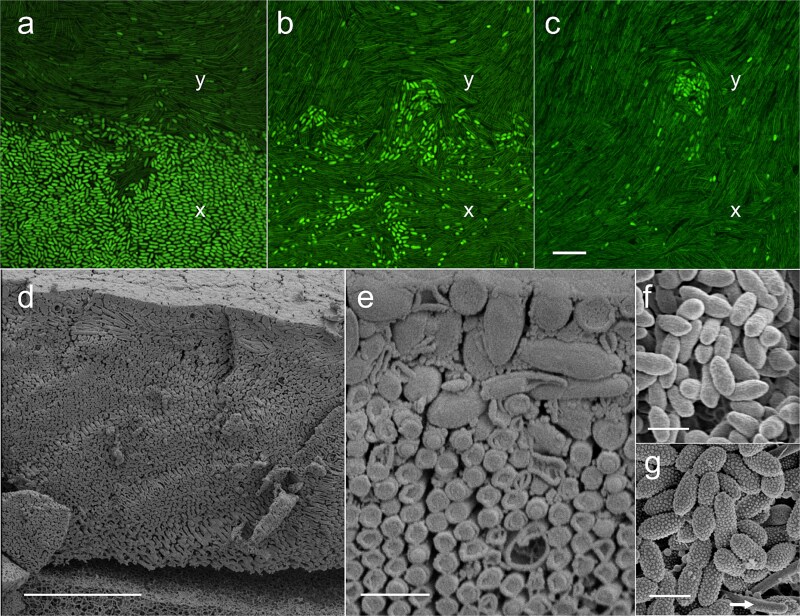
Imaging of C. *l*ytica PlyA3 and *P. pseudonitzschiae* SW. Panels a to c, confocal microscopy of cells stained with Syto9. Shown are three Z-slices through an expanding colony 24 μm high at the point of imaging, 1.1 cm from the edge, after 2 days on RMAR agar. (a) Z-slice through the upper section of the colony, 21 μm above the agar surface. The upper surface of the region imaged was covered in cells of *P. pseudonitzschiae*. *Pseudosulfitobacter pseudonitzschiae* cells were visible as bright, rounded, short cells. Elongated, flexible cells of *C. lytica* PlyA3 were visible. (b) As panel a, but 18 μm above the surface of the agar. (c) As panel a, but 3 μm above the agar. Position x has the same xy coordinates in each image. Scale bar in panel c indicates 10 μm for panels a–c. Panels (d) and (e), cross-sectional arrangement of *C. lytica* PlyA2 and *P. pseudonitzschiae* SW in coculture. Cryogenic SEM images of cocultured colony show a highly aligned, thick layer of *C. lytica* cells (round and regular shaped) beneath a disordered, thinner layer of *P. pseudonitzschiae* (oval and irregularly shaped). Scale bar for panel d is 10 μm and E is 1 μm. Panel (f) SEM of cells of *P. pseudonitzschiae* SW grown in monoculture. (g) SEM of coculture, showing ovoid cells of *P. pseudonitzschiae* SW with surface protrusions and a small number of cells of *C. lytica* (arrow). Scale bar in panels f and g indicates 600 nm.

To gain insights into the strain distribution in the coculture, we visualised the expanding colony after 3 days of co-inoculation via confocal microscopy. *Pseudosulfitobacter pseudonitzschiae* SW stained strongly with Syto9 and showed intense fluorescence, with an oval cell shape. *Cellulophaga lytica* PlyA3 was seen as flexible, more elongated rods that stained less intensely with Syto9. The two species were clearly distinguishable (Fig. 4a-c). A sequence of different optical Z-sections of the co-migration area (1.1 cm from the colony edge) was acquired (Fig. 4a-c). In images of the upper section of the expanding colony the nonmotile *P. pseudonitzschiae* SW cells were visible, largely on top of the gliding *C. lytica* PlyA3 cells. This was confirmed by cryo-SEM (Fig. 4d and e), which showed *Pseudosulfitobacter pseudonitzschiae* SW on top of organised, hexagonally packed cells of *C. lytica*. *Pseudosulfitobacter pseudonitzschiae* SW formed an upper layer, expanding towards the colony boundary, resembling a radial, “dendritic” pattern of spreading previously described for *Pseudosulfitobacter* [42]. This raises the possibility that *C. lytica* PlyA3 was being patterned by the other bacterial species. The cell density of *P. pseudonitzschiae* SW decreased substantially close to the agar surface, which was dominated by *C. lytica* PlyA3 (Fig. 4b-d). In addition, positions 1 (centre) to 5 (edge) of this colony (Fig. 2a) were imaged by fluorescence confocal microscopy in situ or after recovery. Only nonmotile cells of *C. lytica* PlyA3 were observed when taken from positions 1 to 3. Gliding cells of *C. lytica* PlyA3 cells were observed at positions 4 and 5. Nonmotile *P. pseudonitzschiae* SW were visualized when sampled from positions 1 to 4 this strain was not found at the colony edge (position 5). This is consistent with viable counts from the same region, which suggested *C. lytica* PlyA3 alone made up the leading edge of the colony (Fig. 2b).

In order to track bacterially sized objects within the colony, fluorescently labelled, microbeads of a similar size to the bacteria (0.5 μm diameter) were added during inoculation on RMAR agar plates and the colonies imaged after 1–3 days (Fig. S1). Bead dispersal was not observed by *C. lytica* PlyA3 in monoculture; despite the outwards motility of the gliding cells the beads did not leave the inoculation point. As previously noted, the *P. pseudonitzschiae* colony did not expand, and so the co-inoculated beads remained at the inoculation point. However, in the expanding colony of the two strains in coculture, microbeads could be detected up to 1 cm from the starting point after 18 h. The beads colocalized with the dendritic structures of *P. pseudonitzschiae* SW (Fig. S1). This suggests that both bacterial strains are required to move bacterially sized objects across the agar surface and implies binding of the beads of bacteria. Dendritic spreading phenotypes are found in members of this species [42]. These results, taken together, suggest the active transport of the nonmotile *P. pseudonitzschiae* SW by the *C. lytica* consistent with hitchhiking on top layers of gliding *C. lytica* PlyA3.

### *Pseudosulfitobacter pseudonitzschiae* alters the cell surface in response to coculture with *Cellulophaga lytica* PlyA3

Cells from both monoculture and cocultures were examined by SEM. It was notable that *P. pseudonitzschiae* SW formed ovoid cells with a smooth surface when cultivated in monoculture (Fig. 4f). In contrast, cells of *P. pseudonitzschiae* showed a highly textured surface when in coculture (Fig. 4g). This suggests significant changes in cell morphology in *P. pseudonitzschiae* triggered by coculture with *C. lytica* PlyA3. *Cellulophaga lytica* appeared unchanged in morphology in all experiments.

### *Cellulophaga lytica* PlyA2 has a profound effect on *Pseudosulfitobacter pseudonitzschiae* SW metabolism

**Figure 5:**
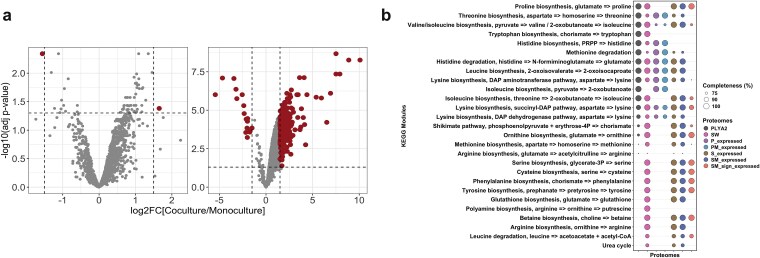
Comparative proteogenomics. (a) Differential abundance of *C. lytica* PlyA2 (left) and *P. pseudonitzschiae* SW (right) proteomes when grown as mono- and coculture. Differentially abundant (|log_2_FC| ≥ 1.5 and adj. *P* ≤ 0.05) proteins between mono- and coculture conditions are depicted as dark red dots and non-differentially abundant (adj. *P* > 0.05) proteins as grey dots. (b) Completeness of metabolic pathways (KEGG modules) associated with the AA metabolism annotated in the theoretical and expressed proteomes under different conditions. Colours of circles indicate the theoretical proteome of *P. pseudonitzschiae* SW (SW) and *C. lytica* PlyA2 (PLYA2), the expressed proteomes in the monocultures of SW and PLYA2 (S_expressed, P_expressed), the expressed proteomes in the cocultures (SM_expressed, PM_expressed) and the significantly different proteomes in the cocultures (adj. *P* ≤ 0.05) (SM_sign_expressed, PM_sign_expressed). Size of circles show the completeness (%) of the metabolic pathways. Only modules more than 70% complete are shown here.

To elucidate the molecular mechanisms underlying strain interactions, we performed proteogenomics. Shotgun proteomics on mono- and cocultures identified differentially abundant proteins (|log_2_FC| ≥1.5, adj. *P*≤0.05) (Table S3). Comparison of the theoretical and expressed proteomes [43] showed 57.3% and 56.1% coverage (expressed/theoretical proteome%) (Table S4). We identified 2400 *P. pseudonitzschiae* SW proteins, with 270 differentially abundant in coculture with *C. lytica* PlyA2 (Fig. 5a). Of these, 253 (93.7%) *Pseudosulfitobacter* proteins significantly increased their abundance. In the case of *C. lytica* PlyA2, only 2 of 1935 proteins were significantly altered in the presence of *P. pseudonitzschiae* SW (Fig. 5a).

Given the large number of differentially abundant proteins, further inspection of their potential functions was done by Clusters of Orthologous Genes (COG) categories assignment (Table S5). In this analysis, we considered as “significantly different proteome” (Fig. S2 and S3), all proteins that were significantly different in abundance between the compared conditions (adj. *P*≤, all were assigned to a COG category, including proteins with log_2_FC < 1.5. A large fraction of the *P. pseudonitzschiae* SW proteome had unknown function (20%) or lacked COG assignment (7%) (Table S5). Most significantly different proteins were associated with amino acid (AA) transport and metabolism (representing 49% of the theoretical proteins in this category), transcription (30%) and energy production and conversion (52%). Proteins involved in translation, ribosomal structure and biogenesis were highly represented (57%). Similarly, proteins related to lipid and secondary metabolite metabolism (COG category IQ) were also amongst the most significantly different proteins with a high coverage of 56% (Table S5).

Kyoto Encyclopedia of Genes and Genomes (KEGG) over-representation analysis highlighted pathways related to AA metabolism, including phenylalanine, tryptophan, and branched-chain AAs (Fig. S4 and Table S6). We further assessed the effect of the coculture on the AA metabolism of *P. pseudonitzschiae* SW by KEGG module analysis (Fig. 5b and Table S7). The presence of *Cellulophaga* appeared to enhance the biosynthesis of eight AAs (proline, lysine, serine, cysteine, phenylalanine, ornithine, tyrosine, and betaine) in *Pseudosulfitobacter* (100% complete and significantly upregulated KEGG modules in the coculture). Notably, *C. lytica* PlyA2 lacked six of these biosynthetic pathways.

Other induced pathways of *P. pseudonitzschiae* SW included ribosome function, carboxylic acids, and quorum sensing (QS) (Fig. S4 and Table S6). QS is the coordination mechanism of gene expression in microbes though intercellular communication with four types of signalling molecules: autoinducer 1 (AI-1, N-acyl-homoserine lactone [AHL]), AI-2, AI-3 and diffusible signalling factor [44]. Genome mining revealed that *P. pseudonitzschiae* SW harboured 10 times more secondary metabolite biosynthetic gene clusters (BGCs) than *C. lytica* PlyA2 (Table S8). Expression data indicated over 50% of proteins for three *Pseudosulfitobacter* BGCs (betalactone, type III polyketide synthase and terpene) were produced with 43% being induced by *Cellulophaga*. There were eight different BGC types in *Pseudosulfitobacter* with the majority coding for AHLs. Almost 40% of the AHL-biosynthesis proteins were identified in *P. pseudonitzschiae* and 84.8% of them showed significantly higher abundance in the presence of *C. lytica* PlyA2. Autoinducer synthases (LuxI) and autoinducer regulators (LuxR) were found in the genome but not detected by proteomics (Table S8).

**Figure 6:**
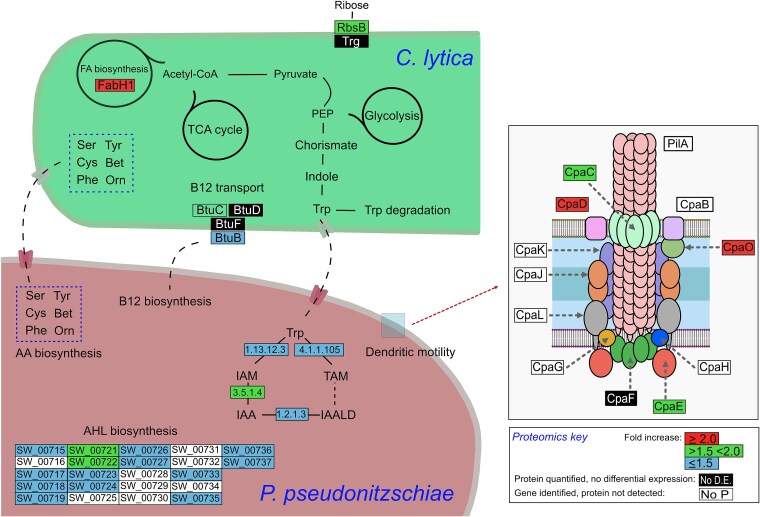
Schematic overview of key metabolic features of C. *l*ytica and *P. ppseudonitzschiae* SW in coculture based on proteogenomics data. The potential for cross-feeding is indicated by dashed black lines connecting the two strains. AAs, for which *C. lytica* was predicted to be auxotrophic and which are produced by *P. pseudonitzschiae*, are shown in dashed blue boxes. The inset for *P. pseudonitzschiae* SW shows the pilus structure thought to be involved in dendritic motility (redrawn from [65]). Proteins are shown on a coloured background relating to proteins found to be significantly upregulated in coculture, as indicated in the proteomics key. Protein names not defined in literature are depicted by their EC number (e.g. 1.13.12.3) or locus tag (e.g. SW_00715). AA, Amino acid; AHL, Acyl-homoserine lactone; Bet, Betaine; Cys, Cysteine; FA, Fatty acids; IAA, Indole-3-acetic acid; IAALD, Indole-3-acetaldehyde; IAM, Indole-3-acetamide; Orn, Ornithine; PEP, Phosphoenol-pyruvate; Phe, Phenylalanine; Ser, serine; TAM, Tryptamine; Trp, Tryptophan; Tyr, Tyrosine.

Besides AHLs, other common, microbially derived signalling molecules are tryptophan and related derivatives, such as indole-3-acetic acid (IAA) [45]. Both strains possessed complete tryptophan biosynthesis pathways (Table S7). *Pseudosulfitobacter pseudonitzschiae* SW may utilize exogenous tryptophan derived from *C. lytica* PlyA2. Key enzymes for tryptophan to IAA conversion via the indole-3-acetamide (IAM) and tryptamine (TAM) pathway were overexpressed in *Pseudosulfitobacter* in coculture (Fig. 6). The induced expression of AHL BGCs and QS-related KOs enriched in the coculture further supports QS involvement by *P. pseudonitzschiae* SW in response to *C. lytica* PlyA2.

Amongst highly differentially abundant *P. pseudonitzschiae* SW proteins (Table S3), a tripartite ATP-independent periplasmic (TRAP) transporter (SW_00684, log_2_FC > 10, adj. p tite ATP-independent periDctP periplasmic protein that binds C4-dicarboxylic acids [46] and a sulfite reductase associated with sulfate assimilation (SW_00614, log_2_FC > 8, adj. *P* ≤ .05) were upregulated. A strong decrease in the abundance was noticed for a phenylacetaldehyde dehydrogenase (SW_02992, log_2_FC < −5, adj. p ≤ adj., which acts in the phenylacetate formation from phenylalanine and a glutamine synthetase (SW_03140, log_2_FC < −4, adj. *P* ≤ C0.05). The glutamine synthase gene was part of an NRPS-like BGC similar to a BGC encoding the antibiotic vicibactin (Table S8).

Another trait of *P. pseudonitzschiae* SW induced in the coculture was spreading in a “dendritic” pattern. To gain more insights on the underlying mechanism, selection of candidate gene clusters was performed according to Bartling *et al.* (2018) [42] followed by genome mining. Three gene clusters potentially responsible for this phenotype were identified, with 12 of 15 associated proteins significantly increased in the coculture (Table S9).

### *Pseudosulfitobacter pseudonitzschiae* SW has a limited effect on the *Cellulophaga lytica* proteome

The theoretical proteome of *C. lytica* PlyA2 consisted of 3378 proteins (Table S4), 74% of which were classified into COG categories of known function and 1935 were being produced. The most significantly different in abundance proteins of *C. lytica* PlyA2 in the presence of *P. pseudonitzschiae* SW were related to cell wall, membrane and envelope biogenesis, energy production and conversion, and coenzyme transport and metabolism (Fig. S3). Differential abundance analysis revealed statistically significant increase of one *C. lytica* PlyA2 protein when grown in coculture: a ribose-importing binding protein RbsB (PLYA2_00165) (Fig. 6 and Table S3). Genome mining detected a single BGC responsible for the production of a carotenoid, with 9 out of 14 of its genes being expressed by *C. lytica* PlyA2, both in mono and coculture (Table S8). Moreover, a putative quorum quenching lactonase, belonging to the metallo-β-lactamase family (PLYA2_02133) involved in QS disruption by degrading AHLs, was present but did not change significantly in abundance between the tested conditions.

### The optical appearance of *Cellulophaga l*ytica PlyA2 colonies is influenced by *Pseudosulfitobacter pseudonitzschiae* SW

**Figure 7:**
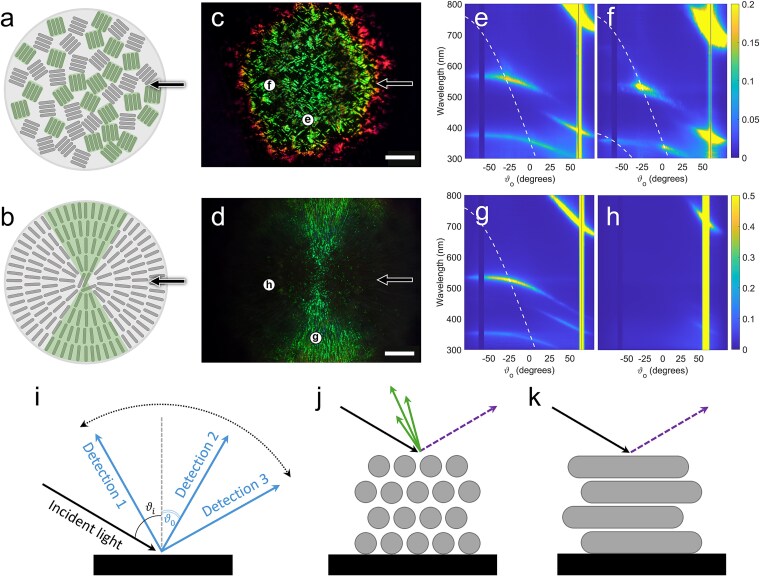
Characterisation of the optical appearance of C. *l*ytica monoculture and coculture with *P. pseudonitzschiae* SW. Panels (a) and (b), schematic of *C. lytica* PlyA2 cell arrangement when in monoculture (a) and when cocultured with *P. pseudonitzschiae* SW (b). Arrows indicate the direction of illumination. Bacterial domains highlighted in green are those whose alignment scatters light at the correct angle, allowing colour to be observed from the top view. In panel (a), bacterial domains are arranged randomly, resulting in a relatively homogeneous colouration with some small dark areas, whereas in panel (b), they are organized radially from the inoculation spot, resulting in a compact disc-like colour appearance. Panels (c) and (d), low-magnification digital micrographs of 1-day old colonies of (c) *C. lytica* monoculture and (d) coculture of *C. lytica* PlyA2 and *P. pseudonitzschiae* SW, both illuminated at an angle of ∼−20° and observed at ∼−25°. Arrows indicate the direction of illumination; letters represent the areas investigated via angle-resolved optical spectroscopy. Scale bars are 2 mm. Panels (e), (f), (g), (h), heatmaps of scattered signal collected in function of wavelength and observation angle. (e) and (f) *Cellulophaga lytica* PlyA2 colonies and (g) and (h) coculture for two different in-plane orientations. (i) Schematic of the angle-resolved optical spectroscopy setup in scattering configuration: The incident light angle ϑ_i_ is fixed, whereas the scattered light is detected at various angles of observation ϑ_o_. (j) Schematic of a hexagonally packed photonic crystal in cross-section, responsible for the angle-dependent scattering patterns shown in panels (e), (f), and (g). This response consists of a specular signal (purple dotted arrow) and scattered light (green arrows). (k) Schematic of multilayer-like periodicity in longitudinal section, which results in the scattering pattern observed in panel k, composed only of a specular signal.

The interaction between these strains influenced the cell ordering, and therefore the optical response of *C. lytica* (Fig. 7a and b)**.** A strong, pointillistic optical response was observed in the *C. lytica* monoculture. This optical appearance is also typical for IR1 [33]. The dark areas do not correspond to lack of growth of the expanding colony or to disordered region but only to different organisation of the local structure which diffract light at angles that cannot be collected by the setup. The colony was organised into a polycrystalline structure, with local domains of aligned cell groups where the bacteria sharing the same orientation were about a hundred microns wide, while the orientation of domains in the x–y plane of the domains was random, resulting in SC seen throughout the colony (Fig. 7c). In contrast, the coculture displayed a radial pattern where there was a much longer-range correlation and the cells were distributed radially across the colony, providing a strong and bright diffraction when the incident illumination light is perpendicular to the orientation of the aligned bacteria (Fig. 7d). To quantify such differences, we performed angle-resolved reflectance spectroscopy in the radially patterned region of the coculture and compared it to the monoculture under two different in-plane rotations.

By illuminating the structure at an angle of incidence ϑi and collecting the scattering response at various angles ϑo (Fig. 7i) we obtained heatmaps (Fig. 7e-h) that show the spectral response in function of the wavelength and observation angle ϑo. Here, we could observe intensity spots at specific wavelengths at angles that are characteristic of the two-dimensional, hexagonal periodic structure typically observed in SC bacteria [33]. Such spots arise from the interference of the incident light with the grating periodicity formed by the bacteria in one direction (Fig. 7j), and their angular distribution can be fitted with the grating equation to retrieve the interbacterial distance [33]. If the optical response is observed for an in-plane rotation of 90°, the grating periodicity is absent and the photonic structure resembles a simple multilayer (Fig. 7k), and therefore the diffraction spots are not observed, confirming the radial orientation of the cells.

Figure 7e corresponds to the angular distribution of the scattered light for ϑ_i_ = −60°. A diffraction spot is observed in the visible range around 530 nm (green) for ϑ_o_ ≃ −30° for *C. lytica* PlyA2, which remains unaltered for a 90° in-plane rotation (Fig. 7f). This was expected from a *C. lytica* PlyA2 monoculture, as the bacteria domains did not have a preferential alignment. In contrast, when *C. lytica* PlyA2 was grown with *P. pseudonitzschiae* SW, a similar diffraction spot was visible only when the incident light was perpendicular to the expansion direction of the colony (Fig. 7g) and was absent for an in-plane rotation of 90° (Fig. 7h). In all cases, the angular distribution of the diffraction spots fitted the grating equation (dotted lines) for the same periodicity of d = 410 nm, which corresponds to the interbacterial distance of *C. lytica* PlyA2 domains. Therefore, the directional grating comes from the radially aligned growth of *C. lytica* PlyA2 underneath the *P. pseudonitzschiae* SW, as observed when imaging the cross-sectional arrangement of the cells via cryo-SEM (Fig. 4d and e). Moreover, we did not observe any specific feature indicating an order in the spatial arrangement of the cells when performing angle-resolved optical spectroscopy on *P. pseudonitzschiae* SW alone. This indicates that *P. pseudonitzschiae* SW contributes to the aligned growth of *C. lytica* PlyA2, changing the colony appearance, but it does not modify the intrinsic two-dimensional, hexagonal periodic structure or its optical response.

## Discussion

Microbial interactions are important for survival and the stability of most ecosystems [47, 48]. Here, we describe a relationship between two strains from the same environment at different levels, including motility, metabolism and cell organisation. Gliding colonies of *Cellulophaga* promoted the spread of the nonmotile *Pseudosulfitobacter*. Under most conditions both species gained, as judged by the association resulting in a greater population of viable cells than axenic culture. However, *P. pseudonitzschiae* SW gained the most and in long term cocultivation on rich medium the viability of the *Cellulophaga* was disadvantaged by the other species, which could be competition for nutrients or a more active antagonism. The *Pseudosulfitobacter* strain was predicted to have diverse secondary metabolite arsenal (Table S8) whose expression was triggered on RMAR (but possibly not to the same extent on RMARLow) by the presence of *Cellulophaga* upon coculture after 3 days. This suggests the beginning of antagonism that escalated by day 10 after co-inoculation. Therefore, the relationship is a dynamic one that can shift from mutualistic to antagonistic depending on the environment.

Gliding *Cellulophaga* transport *Pseudosulfitobacter*, the latter hitchhikes on the former [5]. Gliding bacteria can transport nonmotile bacteria as cargo [8]. The *Roseobacteraceae* (former *Rhodobacteraceae*) are known as exemplary hitchhikers with a versatile physiology to accommodate their needs [45, 50, 52]. Their ecological flexibility is connected to their lifestyle, which includes switching between surface-attached and free-living states, “swim-or-stick”. Recent studies suggest that this biphasic model is QS-regulated by the production of AHLs, which has been linked to attachment on surfaces [50]. QS systems have been reported as conserved within the *Pseudosulfitobacter* genus [53], while AHL signalling molecules have been measured in their cultures [50, 53]. Here, *P. pseudonitzschiae* SW had four gene clusters for AHL biosynthesis with most of the respective proteins being produced, and often significantly increased in the coculture. This suggests a potential role of its QS system in their “hitchhiking” interaction. Additionally, *Cellulophaga* could only transport microbeads when *Pseudosulfitobacter* was present (Fig. S1), which may indicate some form of triggered adhesion mechanism. Release of AHLs by the diatom-dwelling *P. pseudonitzschiae* enhanced biofilm formation and the ability to attach to exopolymeric materials during their “sticky” lifestyle mode [50]. Similarly, it could be that *P. pseudonitzschiae* SW have AHL-regulated adherent properties. It is notable that the two strains formed layers with the *C. lytica* beneath, at the agar surface. This is logical, as *C. lytica* needs to contact the agar to glide. Another gliding *Flavobacterium*, strain IR1, has been shown to “undercut” colonies of adjacent bacteria during predation of the latter maintaining agar contact with the prey bacteria above [20].

*Pseudosulfitobacter pseudonitzschiae* SW does more within the coculture than be passively dispersed, as seen by the increased expression of translation and ribosomal proteins, suggesting enhanced growth. Coculture with the nonmotile *C. lytica* PlyA4 on RMAR showed an increase in the abundance of both strains, which may be explained by metabolic interactions. In addition, *Pseudosulfitobacter* extensively modified AA uptake and metabolism, implying remodelling metabolic pathways in response to *Cellulophaga*. Nutrient complexity can lead to distinct growth behaviours and drive spatial organisation between cell populations [54, 55]. Here, *C. lytica* formed the basal layer of the shared colony on RMAR growth medium, primarily composed of peptone and yeast extract. Therefore, *P. pseudonitzschiae* SW received nutrients after *C. lytica* and may be responding to alterations in the composition and availability of nutrients and/or secreted products. *Cellulophaga lytica* cannot synthesize six AAs, which must harvest from the medium, and these may become less available for the other species. *Pseudosulfitobacter pseudonitzschiae* SW compensates by increasing AA biosynthesis reflected in the differential abundance analysis results (Fig. 5b, Fig. S2 and S4). It may also be that there is a degree of cross-feeding via AA exchange that stabilises this interspecies interaction [56] and may explain why *C. lytica* gains in the number of viable cells under most growth conditions (Fig. 3). Besides AAs, *C. lytica* lacks key genes for vitamin B12 biosynthesis but overexpressed the vitamin B12-specific outer-membrane receptor (BtuB), possibly relying on the interacting partner for supply (Fig. 6). The *Cellulophaga* proteome remained largely unchanged, highlighting the asymmetric nature of the interaction. By aiding *Pseudosulfitobacter* access to nutrient hotspots, *C. lytica* may increase its own acquisition of complex polysaccharides [21, 57].

Irrespective of nutrient availability, *P. pseudonitzschiae* SW population increases in the presence of *C. lytica*, likely triggered by metabolite cues. IAA, a phytohormone primarily from plant-associated bacteria [58], has been identified as a cross-kingdom signalling molecule in algal-bacterial interactions, mediated by AHLs [59]. In coculture experiments of *P. pseudonitzschiae* and closely related algae, IAA promoted selective interaction with beneficial organisms, enhancing growth for both [45, 59]. Overexpression of tryptophan-dependent IAA biosynthesis enzymes and AHL production in *P. pseudonitzschiae* SW with *C. lytica* suggests a role in their population dynamics (Fig. 6). Our results suggest that tryptophan reserves for IAA production are probably derived by both partners. Furthermore, IAA acts as an antagonist of phenylacetic acid production [60], as seen by the suppression of phenylacetaldehyde dehydrogenase in *Pseudosulfitobacter* coculture. Further studies are needed to determine the effect of this metabolite in microbial interactions. Besides IAA, other growth-stimulating metabolites may include organic acids in the media or produced by the partners. Shibl *et al.* [51] identified metabolites that enabled the growth and attachment of phytoplankton-associated bacteria, including *P. pseudonitzschiae*, such as azelaic acid and suberic acid. Uptake of dicarboxylic acids was corroborated by the response of *P. pseudonitzschiae* SW in the presence of *C. lytica* overexpressing a TRAP transporter for dicarboxylic acids.

Both microorganisms segregate yet affect the organisation of the other. *P. pseudonitzschiae* SW was induced to form dendritic structures, supported by candidate gene clusters and induced production of the respective proteins in coculture (Table S9). *Cellulophaga lytica* coculture altered its SC (Fig. 1) due to modulation of its domains of aligned cells, with the radial patterning observed apparently caused by the dendrites of *P. pseudonitzschiae*. Hitchhiking and pattern formation have also been seen in nonmotile *Escherichia coli* and motile *Acinetobacter baylyi*, the latter spreading via twitching and the former acting as the hitchhiker or cargo forming flower-like patterns in coculture [61]. The lack of proteins involved in SC found to be regulated in coculture suggests physical rather than metabolic interactions underlying SC modulation.

Microbial consortia display a wide variety of interactions between each other, but also with the external environment facilitated by metabolic couplings and occurring at several levels. This work describes an intricate interspecies association by tracking their cell organization and viability, optical phenotypes, genomes and proteomes. Follow-up studies investigating the remarkable properties and complex interrelationships of multiorganism consortia (rather than individual strains), including consideration of this system in the field of active matter [62, 63], would enhance our understanding on the multiple types of interactions that exist in nature.

## Supplementary Material

Table_S2_R1_ycaf118

Table_S3_ycaf118

Table_S5_ycaf118

Table_S6_ycaf118

Table_S7_ycaf118

Table_S8_ycaf118

Table_S9_ycaf118

04_Gavriilidou_et_al_ISMECOMMUN_D_25_00059_SI_final_ycaf118

## Data Availability

The data and codes to reproduce the analysis and visualisation underlying this article are available at doi:10.5281/zenodo.12759532. Genomic data for *P. pseudonitzschiae* SW is available at ENA under accession PRJEB77674. The mass spectrometry proteomics data have been deposited to the ProteomeXchange Consortium via the PRIDE [64] partner repository with the dataset identifier PXD053874.
