# Supplementary material for: Pseudosulfitobacter pseudonitzschiae hitchhikes on gliding colonies of Cellulophaga lytica
Source: ISME Commun. 2025 Jul 16;5(1):ycaf118. doi: 10.1093/ismeco/ycaf118 (PMC12376047; doi:10.1093/ismeco/ycaf118)
Supplement: 04_Gavriilidou_et_al_ISMECOMMUN_D_25_00059_SI_final_ycaf118 [file 04_gavriilidou_et_al_ismecommun_d_25_00059_si_final_ycaf118.pdf]

Pseudosulfitobacter pseudonitzschiae hitchhikes on gliding colonies of *Cellulophaga lytica*

Asimenia Gavriilidou[a], Maria Murace[b], Marina Portoghese[b], Sanne Schouten[c], Raditijo Hamidjaja[c], Álvaro Escobar Doncel[c], Sjef Boeren[d], Marcel Giesbers[e], Jérémie Capoulade[f], Silvia Vignolini[b,g], Hauke Smidt[a], Colin J Ingham[g].

[a]Laboratory of Microbiology, Wageningen University & Research, Stippeneng 4, 6708WE, Wageningen, The Netherlands

[b]Yusuf Hamied Department of Chemistry, University of Cambridge, CB2 1EW, United Kingdom

[c]Hoekmine BV, Verenigingstraat 36, 3515GJ, Utrecht, The Netherlands.

[d]Laboratory of Biochemistry, Wageningen University & Research, Stippeneng 4, 6708WE, Wageningen, The Netherlands

[e]Wageningen Electron Microscopy Centre, Droevendaalsesteeg 1, 6708PB, Wageningen, The Netherlands.

[f]Department of Bionanoscience, Kavli Institute of Nanoscience, Delft University of Technology, van der Maasweg 9, 2629HZ, Delft, The Netherlands.

[g]Sustainable and Bio-inspired Materials, Max Planck Institute of Colloids and Interfaces, Potsdam 14476, Germany.

Corresponding authors: Asimenia Gavriilidou (asimenia.gavriilidou@wur.nl); Stippeneng 4, 6708WE, Wageningen, The Netherlands; and Colin J. Ingham (colinutrecht@gmail.com); Am Mühlenberg 1 OT Golm, Potsdam 14476, Germany.

Supplementary Information

Supplementary Methods

Selective viable counts

Whole colony selective viable counts were obtained by harvesting the contents of an entire plate into 20 ml of 3% (w/v) sea salts and plating of 10-fold serial dilutions on RMAR containing rifampicin (25 µg/ml) to selectively isolate *C. lytica* PlyA3 or PlyA4 (Table S1). Clindamycin was used (10 µg/ml) in RMAR agar to select for *P. pseudonitzschiae* SW. Mapping colonies was performed by obtaining agar plugs from different positions in a colony using a sterile, 3 mm diameter cork borer, diluting the bacteria from each sample into 10 ml of sea salts and performing selective viable counts as described above.

DNA sequencing and strain identification

Isolates were identified by sequencing of PCR-amplified variable regions of 16S rRNA genes and comparing with the RDP2 database as previously described [1]. DNA extraction and whole genome sequencing of *C. lytica* PlyA2 was performed by Zomer et al. [2] and data has been deposited under accession PRJEB56913 at the European Nucleotide Archive (ENA). For *P. pseudonitzschiae* SW, DNA extraction was performed using the same protocol as described by Gavriilidou et al. [3] and the genome was sequenced by Novogene (Europe) using the Illumina NovaSeq 6000 PE150 platform.

Genome analysis

Quality filtering of raw reads was done with fastp 0.23.2 [4], and genome assembly of filtered reads followed using SPAdes 3.15.2 [5] (--isolate mode). Only contigs of >500 bp length were considered for downstream analysis and the rest were filtered out with the 'reformat' script of BBTools 38.84 [6]. Assembly quality was assessed by QUAST 5.0.2 [7], and completeness and contamination were estimated by CheckM 1.1.2 [8] using default settings. Prokka 1.14.6 [9] was employed for annotation of protein coding genes, addition of locus tags and translation of coding genes to amino acid sequences for both strains. The resulting amino acid sequences were used as input for the assignment of KEGG (Kyoto Encyclopedia of Genes and Genomes) Orthologs (KOs) and metabolic pathway reconstruction by KofamKOALA [10] online platform and EnrichM 0.5.0 *annotate* and *classify* function [11] (default settings). Completeness (%) of metabolic pathways was estimated based on the number of steps found to the number of steps required for a KEGG module to be complete as annotated by EnrichM 0.5.0. The KEGG annotations were initially performed on the genomes, each gene/predicted

protein was matched to expression using locus tags and the resulting set was used to estimate a given module's completeness. Additional functional annotations were generated with the eggNOG-mapper 2.1.12 [12] web server using default settings. The online version of antiSMASH 7.1.0 was used to identify secondary metabolite BGCs applying "relaxed" detection strictness [13].

Proteomics

Strains for proteomics were grown on RMAR agar, as described above but using 1.2% (w/v) agar and omitting nigrosine. Cultivation was performed on 10 x 10 cm plates, and each plate was harvested separately as a biological replicate (four per condition). For *C. lytica* strains and *P. pseudonitzschiae* SW alone or as coculture, the plates were spread with 10^9 colony forming units (cfu) of one or both strains and incubated 2 days before harvesting. Bacteria were suspended in chilled 3% (w/v) sea salts followed by centrifugation (5,000 x g, 10 min) with the cellular pellet being washed twice with sea salt solution and then processed for the cellular proteomics.

Cell lysis and sample preparation for proteomics

Cell suspensions were pelleted by centrifugation at 5,000 x g and washed twice with 500 µl of 100 mM Tris HCl (pH 8). Cell pellets were resuspended using the same buffer and disrupted by 5-min sonication using a Q500 sonicator (Qsonica, Connecticut, USA) with amplitude of 30% for 2 s and a 2 s pause while cooling on ice. To remove cell debris, the lysates were centrifuged at 10,000 x g at 4°C for 10 min. Protein concentrations of the supernatants were measured with a Qubit Protein and Protein Broad Range (BR) Assay kit (ThermoFisher Scientific, Germany) using a DS-11 FX Fluorometer (DeNovix, USA). A modified version of the protein aggregation capture (PAC) method on microparticles was used for the proteomics sample preparation [14, 15].

Protein identification and quantitation was done by nano liquid chromatography tandem mass spectrometry (nanoLC-MSMS). Peptides were separated with high resolution using a reversed phase nano LC (Thermo nLC1000, Thermo Scientific, MA, USA) equipped with in-house prepared packed 250*0.1 mm columns [ReproSil-Pur Basic C18 1.8 µm beads (Dr. Maisch, Germany)]. MS and MSMS spectra of the peptides were measured with an Orbitrap Exploris 480 (Thermo Scientific, MA, USA) as described by Feng et al. [16].

Proteomics data processing, analysis and visualisation

Raw LCMS-MS data were analysed with MaxQuant 2.0.3.0 [17] using the "Specific Trypsin/P" digestion mode with maximally 2 missed cleavages and the Andromeda search engine with default settings [18]. Modifications, quantification and filtering options were set as reported before [16]. The protein databases included the predicted amino acid sequences derived from the translation of the draft genomes of *C. lytica* PlyA2 [2] and *P. pseudonitzschiae* SW (sequenced and assembled here), which were used together with a database with common contaminants [16]. nanoLC-MSMS system and data quality was checked with PTXQC [19] using the MaxQuant result files.

Label-free quantification (LFQ) intensity values of the filtered protein groups from MaxQuant were used for downstream analysis in R 4.3.2 [20] and RStudio 2023.9.0.463 [21]. Data inspection and quality control (QC) was performed according to the QC workflow of protti R package [22]. Data preparation included log2 transformation of the LFQ intensities using the dplyr R package [23]. Before proceeding with any statistical inference, the type of missingness was assigned to each comparison to check if the defined minimum number of observations per comparison was satisfied with the "assign_missingness" function of the protti R package [22]. When the conditions were at least 70% complete (adjusted downward), missing values were considered 'missing at random' (MAR). Values were considered 'missing not at random' (MNAR) when one condition was complete and the other had less than 20% of the values present (adjusted downward). If all values were present for every replicate of the condition pair, comparison was 'complete'. Conditions with too few observations were not assigned into any of the above categories ('NA') [22]. The same package was used for differential abundance analysis ("calculate_diff_abundance" function) applying a "moderated t-test" based on the *limma* R package [24]. Values assigned to MNAR and NA types of missingness were filtered out prior to p-value adjustment (BH) and thus were not considered for downstream analysis. The data was further subjected to KEGG pathway over-representation analysis using the "enrichKEGG" function of the clusterProfiler R package [25, 26]. Comparisons of the expressed proteomes concerned growth in mono- versus cocultures for *C. lytica* PlyA2 and *P. pseudonitzschiae* SW. Visualization of the data analysis output was performed with enrichplot and ggplot2 R packages [23, 27]. In all cases, differences in protein abundance were deemed significant if $|\log_2 FC| \geq 1.5$ and adj. $p \leq 0.05$.

Supplementary References

1. Hamidjaja R, Capoulade J, Catón L *et al.* The cell organization underlying structural colour is involved in flavobacterium ir1 predation. *ISME J*. 2020;**14**:2890-900 https://doi.org/10.1038/s41396-020-00760-6
2. Zomer A, Ingham CJ, von Meijenfeldt FAB *et al.* Structural color in the bacterial domain: The ecogenomics of a 2-dimensional optical phenotype. *PNAS*. 2024;**121**:e2309757121 https://doi.org/doi:10.1073/pnas.2309757121
3. Gavriilidou A, Gutleben J, Versluis D *et al.* Comparative genomic analysis of *Flavobacteriaceae*: Insights into carbohydrate metabolism, gliding motility and secondary metabolite biosynthesis. *BMC Genomics*. 2020;**21**:569 https://doi.org/10.1186/s12864-020-06971-7
4. Chen S. Ultrafast one-pass fastq data preprocessing, quality control, and deduplication using fastp. *Imeta*. 2023;**2**:e107 https://doi.org/10.1002/imt2.107
5. Prjibelski A, Antipov D, Meleshko D *et al.* Using SPAdes de novo assembler. *Curr Protoc Bioinformatics*. 2020;**70**:e102 https://doi.org/10.1002/cpbi.102
6. Bushnell B, Rood J, Singer E. BBMerge - accurate paired shotgun read merging via overlap. *PLoS One*. 2017;**12**:e0185056. https://doi.org/10.1371/journal.pone.0185056
7. Mikheenko A, Prjibelski A, Saveliev V *et al.* Versatile genome assembly evaluation with QUAST-LG. *Bioinformatics*. 2018;**34**:i142-i50 https://doi.org/10.1093/bioinformatics/bty266
8. Parks DH, Imelfort M, Skennerton CT *et al.* CheckM: Assessing the quality of microbial genomes recovered from isolates, single cells, and metagenomes. *Genome Res*. 2015;**25**:1043-55 https://doi.org/10.1101/gr.186072.114
9. Seemann T. Prokka: Rapid prokaryotic genome annotation. *Bioinformatics*. 2014;**30**:2068-9 https://doi.org/10.1093/bioinformatics/btu153
10. Aramaki T, Blanc-Mathieu R, Endo H *et al.* KofamKOALA: KEGG ortholog assignment based on profile HMM and adaptive score threshold. *Bioinformatics*. 2020;**36**:2251-52 https://doi.org/10.1093/bioinformatics/btz859
11. Boyd JA, Woodcroft BJ, Tyson GW. Comparative genomics using enrichm. In preparation.
12. Cantalapiedra CP, Hernández-Plaza A, Letunic I *et al.* eggNOG-mapper v2: Functional annotation, orthology assignments, and domain prediction at the metagenomic scale. *Mol Biol Evol*. 2021;**38**:5825-29 https://doi.org/10.1093/molbev/msab293
13. Blin K, Shaw S, Augustijn HE *et al.* AntiSMASH 7.0: New and improved predictions for detection, regulation, chemical structures and visualisation. *Nucleic Acids Res*. 2023;**51**:W46-W50 https://doi.org/10.1093/nar/gkad344
14. Batth TS, Tollenaere MX, Rüther P *et al.* Protein aggregation capture on microparticles enables multipurpose proteomics sample preparation. *Mol Cell Proteomics*. 2019;**18**:1027a https://doi.org/10.1074/mcp.TIR118.001270
15. Huijboom L, Rashtchi P, Tempelaars M *et al.* Phenotypic and proteomic differences in biofilm formation of two *Lactiplantibacillus plantarum* strains in static and dynamic flow environments. *Biofilm*. 2024;**7**:100197 https://doi.org/10.1016/j.bioflm.2024.100197
16. Feng Y, Bui TPN, Stams AJ *et al.* Comparative genomics and proteomics of *Eubacterium maltosivorans*: Functional identification of trimethylamine methyltransferases and bacterial microcompartments in a human intestinal bacterium with a versatile lifestyle. *Environ Microbiol*. 2022;**24**:517-34 https://doi.org/10.1111/1462-2920.15886

17. Cox J, Mann M. MaxQuant enables high peptide identification rates, individualized p.p.b.-range mass accuracies and proteome-wide protein quantification. *Nat Biotechnol*. 2008;**26**:1367-72 https://doi.org/10.1038/nbt.1511
18. Cox J, Neuhauser N, Michalski A *et al.* Andromeda: A peptide search engine integrated into the MaxQuant environment. *J Proteome Res*. 2011;**10**:1794-805 https://doi.org/10.1021/pr101065j
19. Bielow C, Mastrobuoni G, Kempa S. Proteomics quality control: Quality control software for MaxQuant results. *J Proteome Res*. 2016;**15**:777-87 https://doi.org/10.1021/acs.jproteome.5b00780
20. R Core Team. R: A language and environment for statistical computing [Computer software]. R Foundation for Statistical Computing. https://www.R-project.org/. 2023.
21. RStudio Team. RStudio: Integrated development environment for R. [Computer software]. RStudio. PBC, Boston, MA. http://www.rstudio.com/ . 2023.
22. Quast J-P, Schuster D, Picotti P. protti: An R package for comprehensive data analysis of peptide- and protein-centric bottom-up proteomics data. *Bioinform Adv*. 2021;**2** https://doi.org/10.1093/bioadv/vbab041
23. Wickham H. ggplot2: Elegant graphics for data analysis [Computer software]. Springer-Verlag New York. ISBN 978-3-319-24277-4, https://ggplot2.tidyverse.org. 2016
24. Ritchie ME, Phipson B, Wu D *et al. limma* powers differential expression analyses for RNA-sequencing and microarray studies. *Nucleic Acids Res*. 2015;**43**:e47-e47 https://doi.org/10.1093/nar/gkv007
25. Yu G, Wang L-G, Han Y *et al.* clusterProfiler: A package for comparing biological themes among gene clusters. *OMICS*. 2012;**16**:284-87 https://doi.org/10.1089/omi.2011.0118
26. Wu T, Hu E, Xu S *et al.* clusterProfiler 4.0: A universal enrichment tool for interpreting omics data. *Innovation*. 2021;**2** https://doi.org/10.1016/j.xinn.2021.100141
27. Yu G. *enrichplot:* Visualization of functional enrichment result. R package version 1.24.4 [Computer software]. https://yulab-smu.top/biomedical-knowledge-mining-book/. 2024.

Supplementary Figures

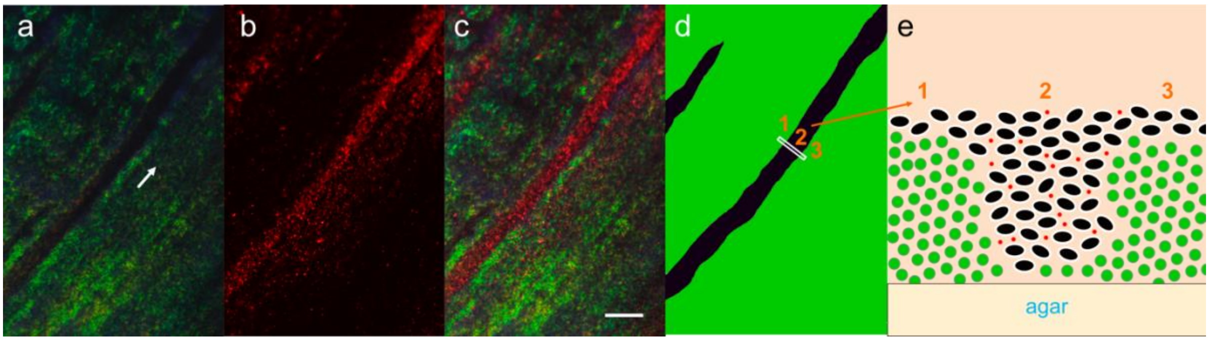

Figure S1 Fluorescent bead dispersal by a cocultured colony of *C. lytica* PlyA3 and *P. pseudonitzschiae* SW. (a) Imaging by microscopy of a location 1 cm away from the inoculation point, using side illumination to view *C. lytica* structural colour (green). The radial striated patterning (see Figure 1) are dendrites of *P. pseudonitzschiae* which exclude *C. lytica*. The arrow indicates the direction of colony expansion. (b) As panel a, but imaging fluorescent microbeads (red) that were inoculated with the bacteria and have been moved to this location. (c) Merged panels a and b, showing the beads largely co-localize with the dendrites. (d) Illustration of the region shown in the previous panels. Black areas represent the two most prominent dendritic processes of *P. pseudonitzschiae* in the field of view (e.g. location 2, also shown in panel e in cross section) and green areas the regions predominantly composed of *C. lytica* with a small number of layers of *P. pseudonitzschiae* on top (locations 1 and 3 in this panel and the next, as seen in Figure 4d). (e) Cross-section of the cocultured colony showing cells of *P. pseudonitzschiae* (black, predominantly in position 2) in a dendritic mass and *C. lytica* (green, positions 1 and 3) with fluorescent beads shown as red spheres. The scale bar in panel c indicates 150 µm for panels a-d. Panel e is not to scale.

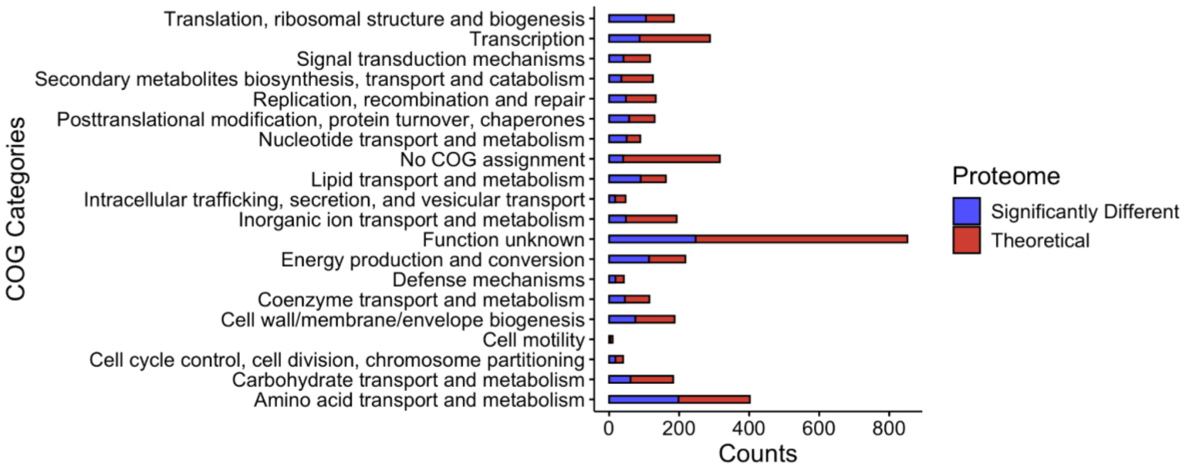

Figure S2. Distribution of proteins into COG categories of the theoretical and significantly different proteome of *P. pseudonitzschiae* SW in mono- vs coculture. Bars represent protein counts as part of the predicted and significantly different in abundance (adj. p≤0.05) proteome of *P. pseudonitzschiae* SW when grown together with *C. lytica* PlyA2 assigned to COG categories. Proteins classified in two-letter COG categories are shown in Table S5.

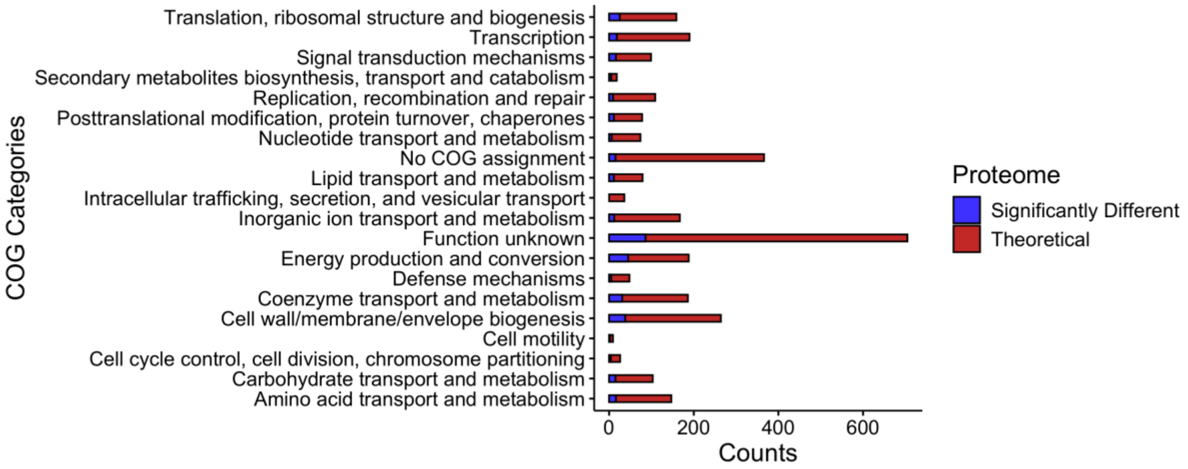

Figure S3. Distribution of proteins into COG categories of the theoretical and the significantly different proteome of *C. lytica* PlyA2 in mono- vs coculture. Bars represent protein counts as part of the predicted and significantly different in abundance (adj. p≤0.05) proteome of *C. lytica* PlyA2 when grown together with *P. pseudonitzschiae* SW assigned to COG categories. Proteins classified in two-letter COG categories are shown in Table S5.

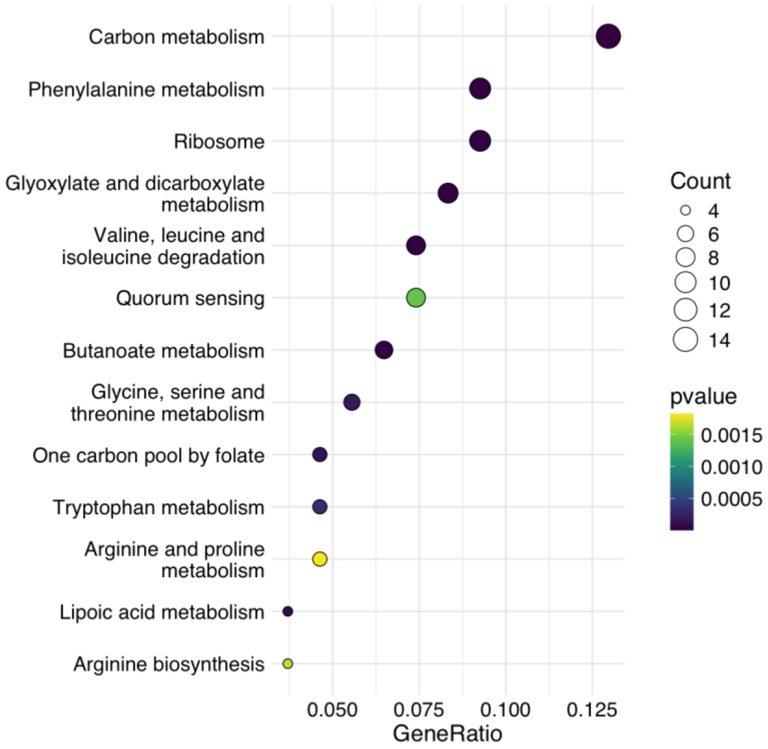

Figure S4. KEGG overrepresentation analysis performed for differentially abundant proteins between *P. pseudonitzschiae* SW mono- and coculture. Dot size indicates the number of KEGG orthologs (KOs) comprising the overrepresented KEGG pathways in the coculture. GeneRatio is the number of KOs belonging to the overrepresented KEGG pathway (counts) and the total number of KOs comprising this pathway in the KEGG database.

Supplementary Tables

Table S1. Strain information.

Species	Strain	Description
Cellulophaga lytica	PlyA2	WT for gliding motility and structural colour
C. lytica	PlyA3	Rifampicin resistant PlyA2
C. lytica	PlyA4	Non spreading mutant of PlyA3
Pseudosulfitobacter pseudonitzschiae	SW	WT isolate, not structurally coloured

Table S2 (Excel File). Supporting data for viable counts. (a) Data supporting Figure 2b and d. Viable counts for colonies of *C. lytica* PlyA3 (Figure 2b), or the non-spreading mutant PlyA4 (Figure 2d), and *P.* pseudonitzschiae SW (indicated as SW) cultured together under different growth conditions after inoculation in the centre of agar plates. Sampling was from different positions, as noted in Figure 2a and c. For each condition selective plate counts were used to calculate the cfu for each strain after plating. The results are expressed as the average cfu per colony for 3 replicates +/- S.D. RMAR, RMAR (0.8% w/v) agar; RMARL (0.8% w/v) agar, low nutrient RMAR plates in 10 x 10 cm Petri dishes. Viable counts were made after 3 or 10 days. (b) Data supporting the viability of *C. lytica* PlyA3 sampled from the colony centre (position 1) in monoculture and in coculture with *P.* pseudonitzschiae SW. The cfu are shown from triplicate assays +/- S.D. Viable counts were made after 10 days of coculture. (c) Data supporting Figure 3. Viable counts for colonies of *C. lytica* PlyA3 and *P.* pseudonitzschiae SW cultured both individually and together under different growth conditions after inoculation in the centre of agar plates. For each condition selective plate counts were used to calculate the cfu for each strain after harvesting the entire colony. The results are expressed as the average cfu per colony for 3 replicates +/- S.D. RMAR, RMAR (0.8% w/v) agar; RMARL (0.8% w/v) agar, low nutrient RMAR plates in 10 x 10 cm Petri dishes. Viable counts were made after 3 or 10 days of coculture.

Table S3 (Excel file). Results of differential abundance analysis of *C. lytica* PlyA2 and *P. pseudonitzschiae* SW proteomes when grown in mono- and coculture. The majority of protein IDs derive from the locus tags of the genes encoding for these proteins.

Table S4. Properties and quality metrics of draft genome assemblies and proteomes of both strains. PlyA2, *C. lytica* PlyA2; SW, *P. pseudonitzschiae* SW.

	***C. lytica* PlyA2**	***P. pseudonitzschiae* SW**
**Genome size (bp)**	3 836 102	4 494 115
**Genes**	3 417	4 329
**Contigs**	48	21
**N50 (bp)**	233 450	485 090
**Completeness (%)**	99.7	99.4
**Contamination (%)**	0.3	0.2
**GC (%)**	32.0	61.8
**Coding DNA sequences (CDS)**	3 378	4 281
**RNAs (rRNAs/tRNAs)**	38	47
**Theoretical proteome**	3 378	4 281
**Expressed proteome**	1 935	2 400
**Coverage (Expressed/Theoretical Proteome %)**	57.3	56.1

Table S5 (Excel file). Abundance of predicted (theoretical) and significantly differently abundant (adj. $p \leq 0.05$) *P. pseudonitzschiae* SW proteins in the coculture assigned to COG categories.

Table S6 (Excel file). KEGG overrepresentation analysis performed for differentially abundant proteins ($|log2FC| \geq 1.5$, adj. $p \leq 0.05$) in *P. pseudonitzschiae* SW cells when in monoculture and coculture with *C. lytica* PlyA2.

Table S7 (Excel file). KEGG module completeness (%) of theoretical (PLYA2, SW) and expressed proteomes under different conditions (mono- vs coculture). PLYA2, *C. lytica* PlyA2; SW, *P. pseudonitzschiae* SW; *_expressed, expressed proteome of monoculture (identified by nanoLC-MSMS); *M_expressed, significantly differently expressed (adj. $p \leq 0.05$) proteomes in coculture.

Table S8 (Excel file). Biosynthetic gene cluster (BGC) information and results of differential abundance analysis of biosynthetic gene-encoded proteins between the tested conditions for both strains.

Table S9 (Excel file). Candidate genes for dendritic motility of *P. pseudonitzschiae* SW. Locus tags as predicted by Prokka are shown. Annotations using different tools described in the Methods and differential abundance (mono- vs coculture) of the respective proteins are presented. Prediction of potential association to the Tad system and function was based on the annotations and literature research.
